# A preliminary study of hepatocellular carcinoma post proton beam therapy using MRI as an early prediction of treatment effectiveness

**DOI:** 10.1371/journal.pone.0249003

**Published:** 2021-03-23

**Authors:** Shen-Yen Lin, Chien-Ming Chen, Bing-Shen Huang, Ying-Chieh Lai, Kuang-Tse Pan, Shi-Ming Lin, Sung-Yu Chu, Jeng-Hwei Tseng

**Affiliations:** 1 Department of Medical Imaging and Intervention, Chang Gung Memorial Hospital, Linkou, Taoyuan, Taiwan; 2 College of Medicine, Chang Gung University, Taoyuan, Taiwan; 3 Department of Radiation Oncology, Chang Gung Memorial Hospital, Linkou, Taoyuan, Taiwan; 4 Department of Gastroenterology and Hepatology, Chang Gung Memorial Hospital, Linkou, Taoyuan, Taiwan; Fondazione Istituto G.Giglio di Cefalu, ITALY

## Abstract

**Purpose:**

To demonstrate the feasibility of magnetic resonance imaging (MRI) for early prediction of proton beam therapy (PBT) effectiveness in hepatocellular carcinoma (HCC).

**Methods:**

Clinical data of the HCC patients without regional lymph node involvement or distant metastasis who received PBT at this institution between 2014 and 2017 were reviewed. A total of 43 patients were included. Tumor regression pattern after PBT were examined on the basis of follow-up duration. The variables were compared between patients with and without early tumor regression (ETR).

**Results:**

The median follow-up duration was 40 months (range, 9–62 months). The cumulative overall survival rate at 6 months, 1 years and 5 years was 100%, 88.4%, 63.4%, respectively. Child-Pugh class A, local tumor control (LTC), complete response (CR), and ETR were significantly associated with overall survival (*p* < 0.05 each). Of 43 patients, 25 patients (58.1%) reached CR in the PBT-irradiated region. Twelve patients (27.9%) had a partial response and 3 patients (7.0%) had a stationary disease. Three patients (7.0%) developed in-field progression. The LTC rate at 5 years was 93.0%. Of the 25 patients who achieved a CR in the PBT-irradiated region, the median time to CR was 5 months (range, 1–19 months). Twenty-two patients (51.2%) showed ETR of the HCC, while 21 patients (48.8%) showed non-ETR. A significant association was observed between ETR and CR of the HCC after PBT (*p* < 0.001).

**Conclusion:**

The post-PBT MRI follow-up at 3 months is helpful for monitoring therapeutic response. ETR of the HCC predicted a higher rate of CR and was associated with overall survival, which provides more accurate clinical management.

## Introduction

Hepatocellular carcinoma (HCC) is a common cancer worldwide [1–3]. Multiple modalities have been used for local treatment, including surgical resection, transplantation, radiofrequency ablation (RFA), percutaneous ethanol injection, transarterial chemoembolization (TACE), and radiotherapy [4–12].

The role of proton beam therapy (PBT) in the treatment of HCC has evolved recently [13–18]. PBT provides benefit of sparing normal tissues because of the drastic dose fall-off after the Bragg peak, and prevents from radiation-induced liver disease (RILD) compared to X-ray therapy [19–21]. Therefore, PBT had been reported to produce positive outcome in the local treatment of unresectable HCC, with 5-year overall survival and local control rates ranging from 23.5% to 44.6% and 83.3% to 90.2%, respectively [14]. However, unlike surgical resection and RFA, a varied time interval between tumor response and radiotherapy was reported, with mean time to complete response (CR) being 6 months but as long as 21 months [22, 23].

Computed tomography (CT) and magnetic resonance imaging (MRI) are commonly used for post-treatment follow-up to HCC after local-regional therapy. However, MRI has no radiation exposure and offers better contrast resolution compared to CT [24, 25]. Tumor response assessment by MRI for HCC after stereotactic body radiation therapy (SBRT) have been reported [26] but literature exploring the imaging changes of the HCC after PBT is limited. This retrospective study aimed to demonstrate post-PBT early MRI assessment and predicted treatment effectiveness for HCC.

## Materials and methods

### Patients

A total of 70 HCC patients without regional lymph node involvement or distant metastasis who received PBT at this institution between 2014 and 2017 were enrolled. Patients with HCC who were concurrently treated with RFA (n = 2), TACE (n = 22), or hepatic arterial infusion chemotherapy (n = 3) were excluded. In total, 43 patients treated for HCC were included. HCC was diagnosed either by pathologic confirmation (n = 17) or on the basis of typical radiologic findings of arterial enhancement and venous washout on dynamic CT or MRI (n = 26). This study was approved by the institutional review board of Chang Gung Medical Foundation (IRB No. 202001510B0), and the need for informed consent was waived because of the retrospective and anonymous nature of the analysis.

### Proton beam therapy

Procedures for PBT have been reported previously [21, 22]. For radiation therapy planning, patients underwent CT simulation in a supine position with the arms above the head. Dynamic CT images were acquired with 2.5-mm intervals in the treatment position by using CT simulator (Discovery CT590 RT, GE Healthcare, Buckinghamshire, UK). Four-dimensional CT and MRI simulations (Optima MR450w MR system, GE Healthcare, Buckinghamshire, UK) were also conducted to determine the tumor motion and margin. All simulation images were transferred to the Eclipse treatment planning system (Version 13.0; Varian Medical System, Palo Alto, California, USA). The gross tumor volume (GTV) was defined as the enhanced area on CT and MRI images. A clinical target volume (CTV) was contoured as the GTV plus a 5–10 mm margin on serial CT images using the treatment system. The respiratory movement range was calculated using four-dimensional CT and added to the CTV as an internal margin. The CTV homogeneously encompassed with more than 95% and less than 108% of the prescribed dose of the isocenter. Before the treatment initiation for each patient, reliability of the proton beam dose distribution was confirmed using a phantom.

Proton beams were generated using a cyclotron (Sumitomo Heavy Industries, Tokyo, Japan), degraded, and then delivered using a wobbling system. The relative biological effectiveness of protons was set at 1.1. The dose-fractionation schedules were 72.6 Gray equivalents (GyE) in 22 fractions for tumors adjacent to the hepatic portal fissure and gastrointestinal tract, 66 GyE in 10 fractions for tumor away from the gastrointestinal tract. A median total dose of 72.6 GyE in 22 fractions (range, 66–72.6 GyE in 10–22 fractions) was given and the median overall treatment duration was 29 days (range, 12–35 days). Dose-volume histogram analyses were performed for all patients. The dose constraints for organ at risk were as follows: gastrointestinal tract: D_max_ < 65% of the total dose; spinal cord: D_max_ < 33 GyE [27].

### Patient follow-up, image analysis and definition

**Fig 1:**
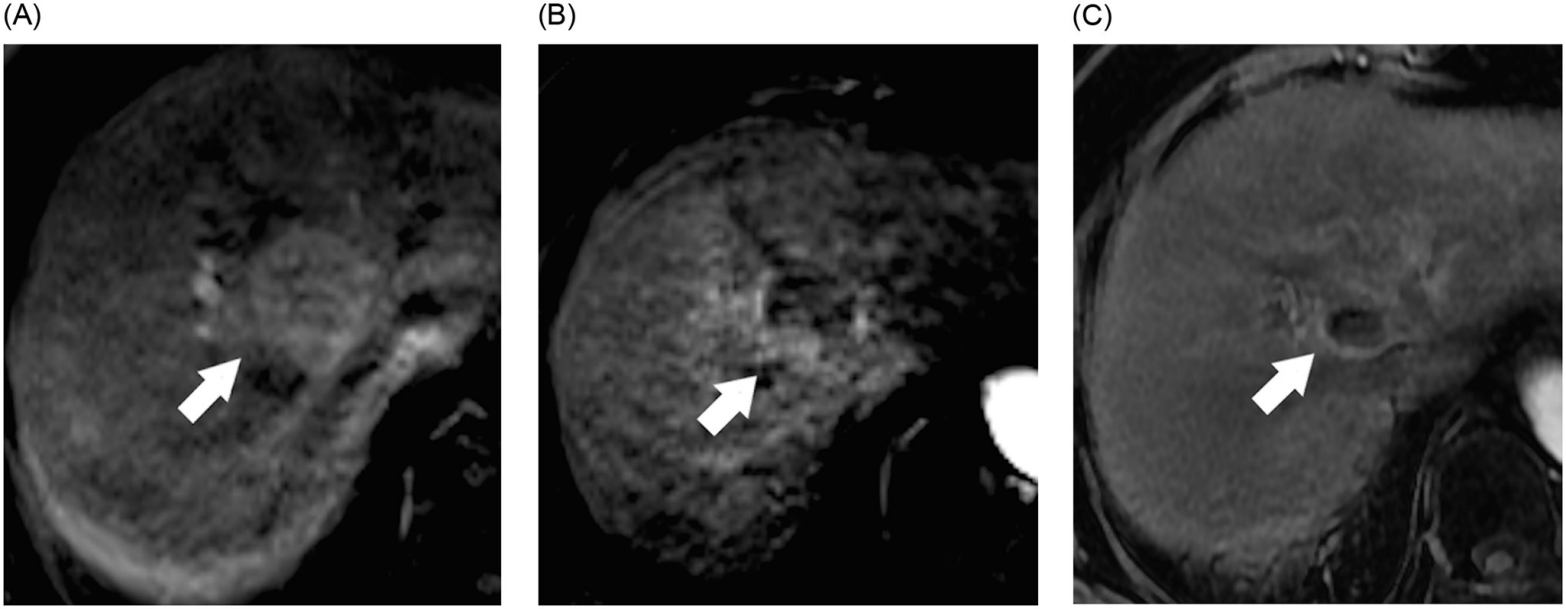
Early tumor regression (ETR) of the hepatocellular carcinoma (HCC) after proton beam therapy (PBT). 74-year-old woman with cirrhosis from chronic hepatitis C virus infection and HCC. MR image obtained before (A), 1 month (B) and 5 months after (C) PBT are shown. (A) Axial T1-weighted image during arterial phase obtained before PBT shows a 3.7 cm HCC in segment 8 (arrow). (B) Axial T1-weighted image during the arterial phase obtained 1 month after PBT shows ETR of the tumor with greater than 50% decrease of diameter of viable tumor (arrow) compared with pretreatment image. (C) Axial T1-weighted image during the arterial phase obtained 5 months after PBT shows lack of enhancement in lesion (arrow); this finding is compatible with complete response.

The patients underwent abdominal MRI studies 1 and 3 months after treatment course completion and then at 3-months intervals. They were evaluated through physical examination and blood tests during the posttreatment follow-up. PBT-related toxicities were evaluated using the Common Terminology Criteria for Adverse Events, Version 4.0. RILD was diagnosed on the basis of both patient symptoms and blood test analysis [28]. Pre- and post-treatment image were performed on 3 Tesla MRI instruments (Magnetom Trio, A Tim System, Siemens, Erlangen, Germany). These included axial fat-saturated T2-weighted sequences, axial diffusion-weighted sequences (b = 800 s/mm^2^), axial in-phase/opposed-phase sequences, and axial gadolinium-enhanced dynamic multiphase sequences. All images were reviewed by 2 radiologists with 7 and 11 years of experience, respectively, with cancer imaging. Each radiologist performed all measurements on the picture archiving and communication system using electronic calipers. They then calculated and recorded the averages of measurement. The tumor response was examined with the modified Response Evaluation Criteria in Solid tumors [29]. A disappearance of any intratumoral arterial enhancement in all target tumors was defined as a CR, a greater than 30% decrease in the sum of diameters of viable (enhancement in the arterial phase) target tumors was defined as a partial response (PR), and an increase at least 20% in the sum of the diameters of viable target tumors within the in-field target volume was defined as progressive disease (PD). A patient was defined as having a stable disease (SD) when they did not qualify for having either a PR or PD. Local tumor control (LTC) was defined as no progression in the irradiated field. Early tumor regression (ETR) was defined as a greater than 50% decrease in the sum of diameters of viable target tumors after PBT and within 3 months on follow-up images compared with pretreatment images (Fig 1).

### Statistical analysis

Survival and disease control rates were calculated at the end of PBT by using the Kaplan–Meier method. Tests for significance of prognostic factors for overall survival and LTC were evaluated by log-rank test. Continuous data were expressed as the mean ± standard deviation. When distributions were skewed, they were expressed as median and interquartile range. A comparison between the ETR and non-ETR groups were performed using a chi-square or Fisher’s exact test for categorical variables. All statistical significances were set at *p* < 0.05, and IBM SPSS Statistics 20 was used for statistical analysis.

## Results

Table 1 lists the demographic, clinical laboratory, and tumor characteristics of the patients. Thirty-six patients had undergone previous therapies involving other treatment modalities, namely surgical treatment (n = 9), TACE (n = 13), RFA (n = 10), and sorafenib (n = 4).

**Table 1:** Patient and tumor characteristics.

Characteristics	N = 43
Age (year), median (range)	71 (48–85)
Sex, male/female	30/13
Performance status, 0/1/2	22/19/2
Child-Pugh classification, A/B	40/3
AJCC, I/II/III	24/10/9
Etiology of liver disease	
HBV	23 (53.4%)
HCV	15 (34.9%)
Alcoholic	2 (4.7%)
Liver cirrhosis	42 (97.7%)
Underlying disease	
Hypertension	21 (48.8%)
Diabetes mellitus	12 (27.9%)
Coronary artery disease	7 (16.3%)
Alfa fetoprotein, ng/mL	
Median	13.2
Range	2.1–143121.3
Tumor size in maximum diameter	
Median (range), cm	3.1 (1.1–17.1)
< 5 cm	31 (72.1%)
≥ 5 cm	12 (27.9%)
Number of tumors	
Single	33 (76.7%)
Multiple	10 (23.3%)
Portal vein thrombosis	
Present	8 (18.6%)
Absent	35 (81.4%)
Hepatic vein thrombosis	
Present	3 (7.0%)
Absent	40 (93.0%)
Bile duct dilatation	
Present	6 (14.0%)
Absent	37 (86.0%)

AJCC: American Joint Committee on Cancer 8^th^ edition; HBV: hepatitis B virus; HCV: hepatitis C virus.

### Outcomes

**Fig 2:**
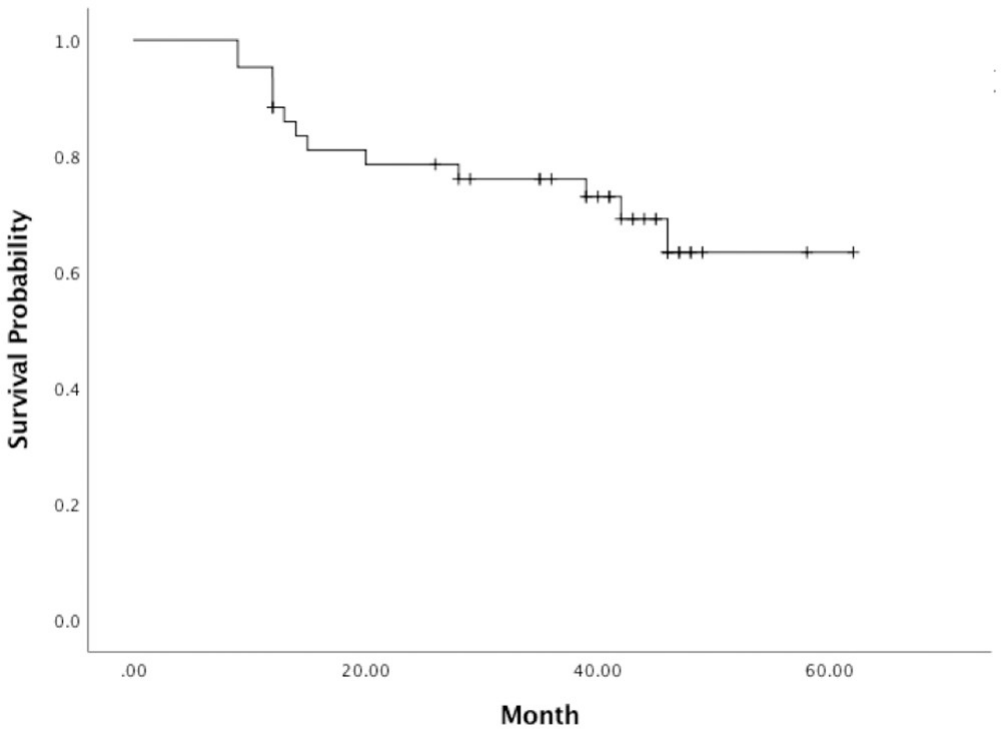
Kaplan-Meier curve of overall survival curve.

The median follow-up duration was 40 months (range, 9–62 months). The cumulative overall survival rate at 6 months, 1 years and 5 years was 100%, 88.4%, 63.4%, respectively (Fig 2). At the time of analysis, 13 patients had died due to empyema (n = 1), bacterial peritonitis (n = 1), pneumonia (n = 2), sepsis (n = 2), stroke (n = 1), intrahepatic tumor progression (n = 1), hepatic failure (n = 4), and respiratory failure (n = 1). Child-Pugh class A, LTC, CR, and ETR were significantly associated with overall survival (*p* < 0.05 each, Table 2).

**Table 2:** Univariate analysis of factors for overall survival.

Variables		N	OS at 5 years (%)	*P* value
Gender	Male	30	0.598	0.979
	Female	13	0.673	
Age	≤ 70 year	19	0.648	0.256
	> 70 year	24	0.609	
Hepatitis B virus	No	20	0.693	0.764
	Yes	23	0.609	
Hepatitis C virus	No	28	0.567	0.699
	Yes	15	0.733	
Hypertension	No	22	0.671	0.754
	Yes	21	0.595	
Diabetes mellitus	No	31	0.641	0.578
	Yes	12	0.635	
Coronary artery disease	No	36	0.850	0.286
	Yes	7	0.833	
Liver cirrhosis	No	1	1.000	0.539
	Yes	42	0.626	
Child-Pugh Classification	A	40	0.669	0.022
	B	3	0	
AJCC	I-II	34	0.592	0.529
	III	9	0.778	
Number of tumors	Single	33	0.603	0.915
	Multiple	10	0.700	
Tumor size	< 5 cm	31	0.668	0.140
	≥ 5 cm	12	0.571	
Portal vein thrombosis	Absent	35	0.650	0.511
	Present	8	0.583	
Hepatic vein thrombosis	Absent	40	0.628	0.866
	Present	3	0.667	
Bile duct dilatation	Absent	37	0.643	0.724
	Present	6	0.625	
Local tumor control	Yes	40	0.683	< 0.001
	No	3	0	
Complete response	CR	25	0.918	< 0.001
	Non-CR	18	0	
Early tumor regression	ETR	22	0.856	0.014
	No-ETR	21	0.381	

AJCC: American Joint Committee on Cancer 8^th^ edition; CR: complete response; ETR: early tumor regression; GyE: Gray equivalents; OS: overall survival.

**Fig 3:**
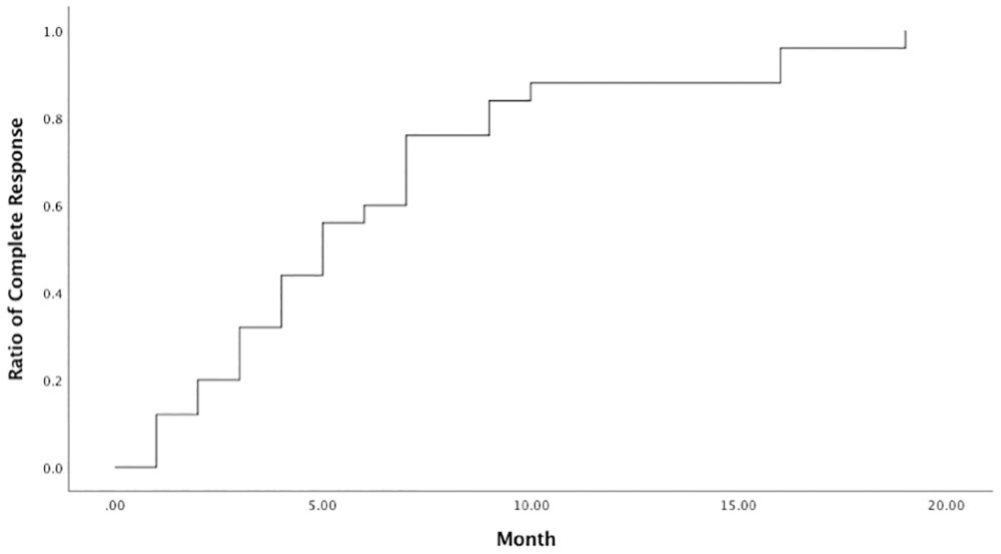
Pattern of the patients with complete response after proton beam therapy. The median time to complete response was 5 months (range, 1–19 months).

Of 43 patients, 25 patients (58.1%) reached CR in the PBT-irradiated region. Twelve patients (27.9%) had a PR and 3 patients (7.0%) had a SD. Three patients (7.0%) developed in-field progression. Of the 25 patients who achieved a CR in the PBT-irradiated region, the median time to CR was 5 months (range, 1–19 months). Nine patients (36.0%) had a CR within 3 months, 15 patients (60.0%) had a CR within 6 months, and 22 patients (88%) had a CR within 12 months (Fig 3).

Of the 43 patients, 3 (7.0%) had infield local progression and 15 (34.9%) had outfield progression (12 had new hepatic tumors, 1 had lymph node metastasis, and 2 had distant metastasis). The LTC rate at 5 years was 93.0%. Dose, target tumor size, number of the tumors, and Child-Pugh classification were not significant factors for LTC. The progression-free survival rates at 1 and 5 years were 74.4% and 55.9%, respectively.

### Tumor regression on MRI

**Fig 4:**
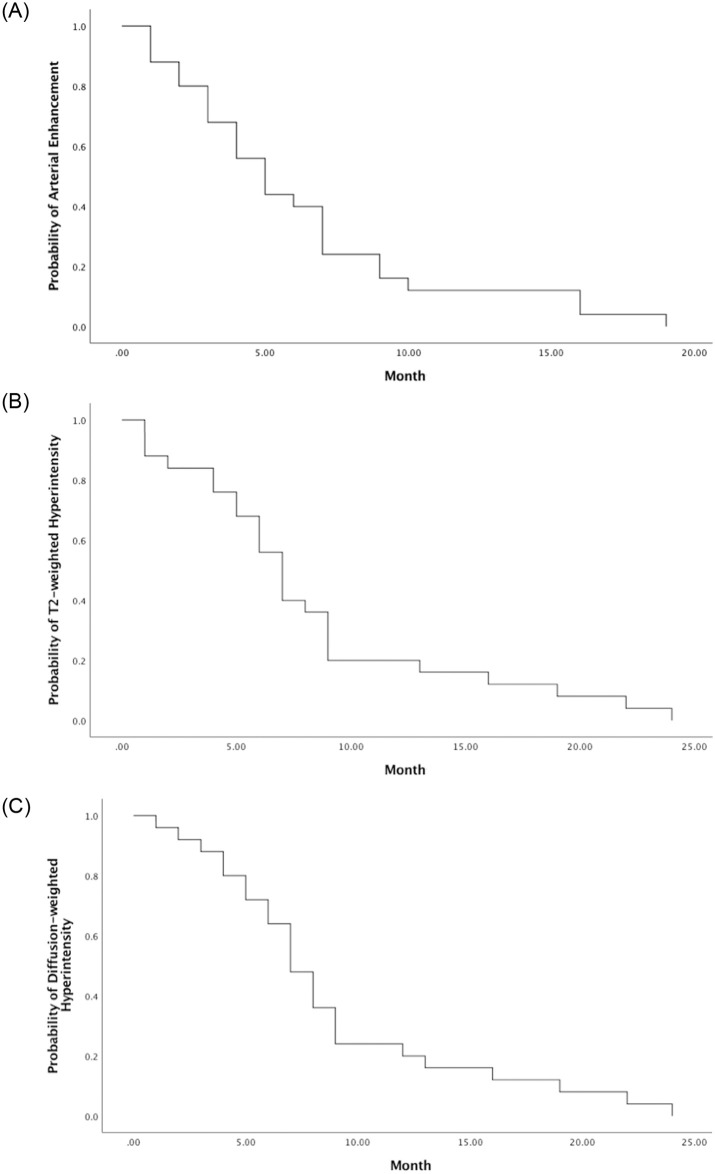
Incidence of tumor regression on magnetic resonance imaging. The incidence of tumorous arterial enhancement (A), T2-weighted hyperintensity (B), and diffusion-weighted hyperintensity (C) of the patients with complete response.

Of the patients with CRs, the tumorous arterial enhancement disappeared completely in 36% (9 out of 25) of the patients at 3 months, 60% (15 out of 25) at 6 months, and 88% (22 out of 25) at 12 months (Fig 4A). The T2-weighted high signal decreased in 16% of the patients at 3 months, 44% at 6 months, and 80% at 12 months (Fig 4B). The diffusion-weighted hyperintensities decreased in 12% of the patients at 3 months, 36% at 6 months, and 80% at 12 months. (Fig 4C). The median time of total disappearance of arterial enhancement, T2-weighted hyperintensity and diffusion-weighted hyperintensity were 5 months, 7 months, and 7 months respectively. Prolonged T2-weighted hyperintensity and diffusion-weighted hyperintensity after complete disappearance of arterial enhancement occurred in 8 and 11 patients, respectively.

### ETR and non-ETR after PBT

Twenty-two patients had ETR and 21 patients had non-ETR. Of the patients with an ETR, 20 (90.9%) had a CR, one had a PR, and one had local progression. Of the patients with ETR, 10 (45.5%) achieved ETR at 1 month. Of the patients without ETR, five had a CR, 11 had a PR, three had SD, and two had local progression.

Table 3 summarizes the patient characteristics, pretreatment image findings, and complete response and mortality between the patients with ETR and non-ETR. The age, gender distributions, and clinical information were similar. Tumor size < 5 cm (*p* = 0.005) was significantly associated ETR. Patients with ETR had a higher rate of complete response of the HCC (*p* < 0.001) and a lower rate of mortality (*p* = 0.015) than patients without ETR.

**Table 3:** Comparison of ETR and non-ETR group.

Variables, n (%)	ETR (n = 22)	Non-ETR (n = 21)	*P* value
Gender (male)	15 (68.2)	15 (71.4)	0.817
Age > 70 year	12 (54.5)	12 (57.1)	0.864
Hepatitis B virus	11 (50.0)	12 (57.1)	0.639
Hepatitis C virus	10 (45.5)	5 (23.8)	0.137
Hypertension	8 (36.4)	13 (61.9)	0.094
Diabetes mellitus	7 (31.8)	5 (23.8)	0.558
Coronary artery disease	5 (22.7)	2 (9.5)	0.412
Liver cirrhosis	22 (100.0)	20 (95.2)	0.488
Child-Pugh Classification			
A	20 (90.9)	20 (95.2)	> 0.999
B	2 (9.1)	1 (4.8)	
AJCC			
I-II	17 (77.3)	17 (81.0)	> 0.999
III	5 (22.7)	4 (19.0)	
Number of tumors			
Single	19 (86.4)	14 (66.7)	0.162
Multiple	3 (13.6)	7 (33.3)	
Tumor size < 5 cm	20 (90.9)	11 (52.4)	0.005
Portal vein thrombosis	6 (27.3)	2 (9.5)	0.240
Hepatic vein thrombosis	3 (13.6)	0 (0)	0.233
Bile duct dilatation	2 (9.1)	4 (19.0)	0.412
Radiation dose			
72.6 GyE	11 (50.0)	14 (66.7)	0.268
66 GyE	11 (50.0)	7 (33.3)	
Complete response	20 (90.9)	5 (23.8)	< 0.001
Local tumor control	21 (95.5)	19 (90.5)	0.607
Mortality	3 (13.6)	10 (47.6)	0.015

AJCC: American Joint Committee on Cancer 8^th^ edition; CR: complete response; ETR: early tumor regression; GyE: Gray equivalents.

### Toxicity

Acute toxicity involving the skin was noted in 22 patients, and 1 of them developed grade 3 toxicity. One patient developed acute grade 1 gastrointestinal toxicity. Seven patients had a Child-Pugh score deterioration of 1 point.

## Discussion

Varied time interval between tumor response and radiotherapy as well as signal changes related to HCC after SBRT and PBT have been reported [21–23, 26, 30]. Kim et al [22] reported the mean time for patients achieving CR was 6.3 months (range, 1–21.7 months), and 93.9% of patients had a CR within 12 months. Kawashima et al [23] reported median time of patients having CR of 8 months (range, 5–20 months). In this study of 25 patients who achieved a CR, 22 (88.0%) exhibited a CR within 12 months after PBT. The median time to CR was 5 months (range, 1–19 months), which is similar to that in previous report. In this study, decreases in the arterial enhancement, T2-wighted hyperintensity, and diffusion-weighted hyperintensity of the tumors after PBT were examined on the basis of follow-up duration. The median time to total disappearance of the arterial enhancement, T2-weighted hyperintensity and diffusion-weighted hyperintensity was 5 months, 7 months, and 7 months, respectively. On the basis of the study findings, it is recommended to monitor the regression of HCC and evaluate the tumor response every 3 months in the first year.

In this study, an ETR was significantly associated with a CR after PBT in patients with HCC. Of the patients with an ETR, 90.9% eventually had a CR and only one patient had local progression. Kim et al [22] reported that the distributions of clinical characteristics were not significantly different between the patients who did and did not have CRs. Fukda et al [31] also reported that no factor significantly affected the local tumor control rate from PBT in patients with HCC. Due to the varied time intervals between tumor responses and radiotherapy, with a mean time of five months but as long as 19 months in this study, and in addition to examining tumor markers and conducting a follow-up with image studies, a response in the first three months might help clinicians better communicate follow-up treatment plans, expected outcomes after PBT and avoid unnecessary biopsy for confirmation of residual or recurrent tumor.

Tumor size was a factor in the success rate of SBRT for HCC, and the patients with large tumor generally had less favorable outcomes [26, 32]. Huang et al reported that the tumor size ≤ 4 cm was an independently significant predictor for higher survival rate [32]. In this present study, a tumor size of < 5 cm was significantly associated with ETR.

In this study, the 5-year overall survival rate was 63.4%. Child-Pugh class A, CR, LTC and ETR were significantly associated with overall survival. Child-Pugh score and tumor response had been reported as prognostic factors for overall survival [22, 33]. The higher 5-year overall survival rate in this study may be reflected by higher numbers of patients with Child-Pugh class A (40/43, 93.0%). MR-guided radiotherapy with hybrid MR-linear accelerator (MR-linac) system is a promising radiation technique, by using MRI for real-time monitoring and dose delivery [34, 35]. Based on this study’s result, MRI for assessment of ETR post MRI-linac may be useful. Hence, the role of MRI in the treatment of HCC will be more crucial.

High and long-lasting local control rates (greater than 80% at 5 years) had been reported [14] with the use of PBT for HCC. However, the rate of new tumor forming outside the treated volume was high, range from 36% to 85% [14]. In this present study, local control rate at 5 year was 93%. Fifteen patients (34.9%) had disease progression outside the irradiated volume after PBT. This means that regular follow-up with image study to find new out-field lesion was required, as was further treatment. Functional imaging modalities, such as 2-deoxy-2-(^18^F)fluoro-D-glucose (^18^F-FDG) and ^11^C-choline Positron-emission tomography (PET)-CT in HCC have made progress. ^11^C-choline has a high PET signal in liver tumor cell and HCC foci gain a better tumor-to-background contrast with choline. ^18^F-FDG PET has a high sensitivity for detecting extrahepatic metastasis but suboptimal sensitivity for local tumor status [36, 37].

This study has a few limitations. First, it was a single center, retrospective, nonrandomized study with a limited number of included patients. Several patients with concomitant local treatment were excluded, in order to reduce confounding factors. Although current data is supportive of early prediction for tumor regression, an ongoing large scale study is needed for further validation. Second, it had an inherent selection bias because some patients were referred for PBT because they refused surgery or conventional local treatment. This is reflected in the higher performance status and lower Child-Pugh scores in this cohort. Current guidelines reserve SBRT and PBT for stage IV palliation [4, 10].

In conclusion, post-PBT MRI follow-up at 3 months is helpful for monitoring therapeutic response. ETR of the HCC predicted a higher rate of CR and was associated with overall survival, which provides more accurate clinical management.
